# Rehabilitation Engineering: A Narrative Review on Recent Advances in Mobility Aids in India

**DOI:** 10.7759/cureus.53722

**Published:** 2024-02-06

**Authors:** Himanshu Raj, Roshan Prasad, Pramita Muntode Gharde, Swarupa Chakole, Prachi Sharma

**Affiliations:** 1 Surgery, Jawaharlal Nehru Medical College, Datta Meghe Institute of Higher Education and Research, Wardha, IND; 2 Internal Medicine, Jawaharlal Nehru Medical College, Datta Meghe Institute of Higher Education and Research, Wardha, IND; 3 Community Medicine, Jawaharlal Nehru Medical College, Datta Meghe Institute of Higher Education and Research, Wardha, IND

**Keywords:** osseointegration, 3-d printing, prosthesis, mobility aids, disability

## Abstract

Mobility has been characterized as the capacity to move across an environment safely, pleasantly, elegantly, and autonomously. India's current population is 1.4 billion, out of which 2.3%, i.e., 32 million people, are suffering from some kind of disability. With the rise in the geriatric population, the incidence of non-communicable and communicable diseases also rises and presents the risk of disorders that may progress to disability. People often neglect their disability and learn to live with it, even when most of them can use rehabilitation programs in conjunction with various mobility aids. Affordable access to adequate healthcare and assistive devices is limited, contributing to the challenges faced by disabled adults. Despite the potential for many disabled individuals to engage in productive work, their employment rates remain significantly lower. Mobility aids can provide significant benefits to individuals affected by a range of medical conditions, including arthritis, cerebral palsy, developmental disabilities, diabetic ulcers and wounds, fractures or broken bones, injuries, and walking impairments resulting from brain injury or stroke. Each person is different and may require help in a certain way for their disability, so choosing the most appropriate aid is crucial for the individual's well-being. Commonly used mobility aids are canes, walking sticks, walkers, and wheelchairs, with prostheses being used less commonly. With the advent of techniques such as state-of-the-art 3D printing and challenging surgeries, various Indian tech companies, along with non-governmental organizations (NGOs), have brought about many significant changes in the world of prosthesis by making it better, affordable, and accessible.

## Introduction and background

**Figure 1:**
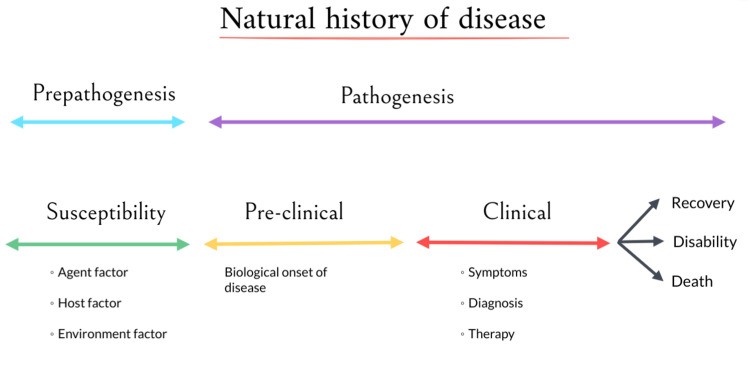
The natural history of disease A flowchart created by the authors plotting the course a disease takes without any treatment, from its inception (pre-pathogenesis), through the various stages of its progression in the pre-clinical and clinical phases, until its resolution, either in the form of recovery, disability, or death [1].

Health is a state of complete physical, mental, and social well-being, not merely the absence of disease or infirmity. If there is any deviation from normal functioning or a state of complete physical or mental well-being, it can be defined as a disease. The disease process is not straightforward but a series of complex interactions between the host, the agent, and the environment, as shown in Figure 1. The progression of any disease, from its initial stage to its eventual outcome, whether it is recovery, disability, or death, in the absence of any intervention or treatment, is referred to as the natural history of the disease [1].

Recovery is the most desirable outcome for an individual affected by any illness. The person is free of any signs and symptoms of the disease and can resume their normal daily life without affecting their quality of life [2]. If no necessary interventions are undertaken during the illness phase of the disease, it might lead to disability. Disability can be defined as a physical or mental condition that limits a person's movement, senses, or activities and makes it difficult to carry out certain activities and interact with the world. Disability can be due to certain conditions present at birth that affect functions later in life, traumatic injuries, and long-standing disorders such as diabetes [3].

**Figure 2:**
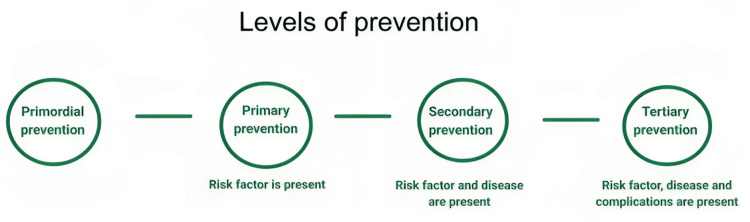
The four levels of disease prevention The flowchart created by the authors describes different stages of prevention at which healthcare interventions can be implemented to prevent the occurrence, progression, or complications of disease [4].

We can undertake certain approaches and activities to reduce the likelihood of diseases and disorders, interrupt or slow the progress of disorders, and reduce disability. There are four levels of prevention strategies (Figure 2), which aim to prevent the onset of disease through risk reduction and reduction of downstream complications of a manifested disease, known as levels of prevention [4].

According to the 2011 census, among the total Indian population of 121 crores (1.21 billion), 2.68 crore (26.8 million) individuals are classified as 'disabled,' accounting for approximately 2.21% of the overall population. Among the major types of disabilities, locomotor disability contributes nearly 20.3%, while hearing and visual impairment account for 18.9% and 18.8%, respectively [5]. A study done by Pattnaik et al. [6], based on secondary data from the National Family Health Survey 2020-2021 (NFHS-5), shows that the overall prevalence of disability among Indians has risen to 4.52% due to an increase in prevalence, in which locomotor disability accounts for 44.70% of all the accounted disabilities, while mental, speech, visual, and hearing disabilities account for 20.28%, 14.55%, 12.02%, and 8.45%, respectively [6].

## Review

Methodology

We conducted a thorough literature search to find pertinent papers for our review article. We searched the databases of PubMed, Scopus, and Google Scholar using the keywords "osseointegration," "3D printing," "prosthesis," "mobility aids," and "disability." Manual searches of reference lists were also carried out to find other pertinent studies. Our inclusion criteria incorporated studies focusing on the types of mobility aids, government and non-government initiatives for the disabled, and challenges in assistive mobility solutions. Original research papers as well as review papers were taken into consideration for inclusion. We included only articles that were written in English, particularly those that were recently published, i.e., between 2010 and 2023, which provide value for comprehensive research. Articles written in languages other than English and those with missing or inaccessible full texts were excluded. Titles, abstracts, and full-text papers were screened during selection. The authors reached a consensus to settle disagreements on the choice of studies.

Mobility and types of mobility aids

Mobility is the ability of an individual to move joints in their body to interact with their surroundings and manipulate objects as per their will. We take our precious gift of mobility for granted until we acquire some special diseases, either congenitally or after birth, which lead to impairments, which further progress to disability and ultimately result in a major disadvantage for an individual [7].

**Figure 3:**
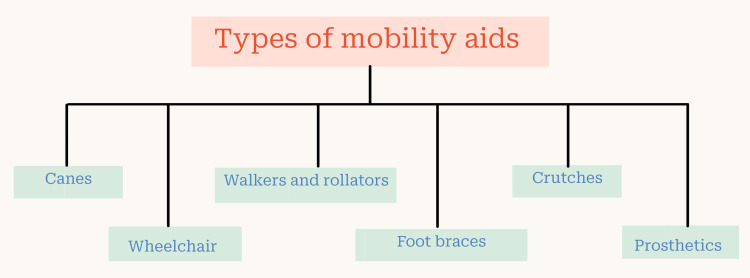
Types of mobility aids Image created by authors

According to the Person with Disabilities Act of 1995, locomotor disability, a major category of physical disability, is defined as the inability of a person to do specific actions linked with the movement of self and things as a result of musculoskeletal or nervous system disease or both. Some common causes of locomotor disability are paralysis, deformity, and loss or dysfunction of the limb. To ease the suffering and lack of mobility, many companies have joined with medical science to bring new advances in mobility aids. Mobility aids allow individuals with difficulty moving to move more flexibly and independently [8]. These devices benefit users by reducing pain (if any), increasing confidence and self-esteem, and providing them with independence of movement. Figure 3 lists the various types of mobility aids that are currently available [8].

Smart Wheelchairs

Standard wheelchairs are merely wheels fitted on the sides of a chair to enable limited independence for the user at the cost of exertion by manually operating the wheels by hand. The wheelchair has limitations when it comes to being operated by any old person or someone who lacks physical strength, and hence there is a need for a helper. This has been overcome with the advent of science and engineering solutions, namely motor-driver wheelchairs and smart wheelchairs. Unlike traditional wheelchairs, these smart machines are now capable of steering efficiently with a single joystick without much physical strength at variable speed, sensing and alerting the person of any obstacle in front of them, sending SMS alerts in case of any malfunctions, and improving operational hours. This has removed the need for added assistance and enables freedom of movement for the user [9].

Canes With Sensory Aid

A long stick, 122 cm in height on average, is used by individuals with visual impairment or those with minor problems with their stability due to pain or injury. The standard cane consists of three parts: the grip, the shaft, and the tip. The tip and shaft work to transfer tactile information to the grip. It transfers the load of that limb onto the cane. Previously made from straight wood, it has now evolved and is manufactured from either aluminum, fiberglass, or carbon fiber. The only problem for the visually impaired person is the fear of unknown obstacles placed above their waistline [10]. Common obstacles like tree branches or sign boards can become a hazard for the visually impaired. It has been tackled by engineering various sensors and sonars to detect obstacles in the path; these devices are easily rechargeable, long-lasting, and universally mountable on any ordinary white cane. An upgrade is currently under development by the Mobility Assistant for Visually Impaired (MAVI) project to not only detect but also describe the obstacle [11]. It is set to enable pedestrian detection, sign board detection, animal detection, text detection, and optical character recognition (OCR) [12].

Walkers and Rollators

Traditional walkers are externally held frames for disabled individuals who require extra assistance to maintain balance or stability when walking. Despite their usefulness, they have significant shortcomings, such as being heavy and laborious to use, often obstructing one's walking, and offering less mobility to the elderly. So, engineers replaced them with a more modern walker that allows greater handling, namely the walkers known as rollators. A walker is a frame with handles and legs that must be raised to move, whereas a rollator has wheels and is pushed. It has several advantages over a conventional walker, as it functions well outdoors due to the wheels fitted on it. They are height-adjustable and more transport-friendly. They also come with an optional seating mount. They are well suited for a person with a movement disability, as they can be designed to meet their specific requirements [13].

Foot Braces

There are different types of foot braces, which are designed to support various parts of the foot, provide for a steady, painless, and efficient gait, and help in fast recovery without hampering the mobility of the patient. Maintaining the foot in a plantigrade position is the primary goal of an ankle-foot orthosis (AFO). In addition to facilitating function, this offers a firm foundation of support that might lessen tone throughout the gait's stance phase. During the swing phase, the AFO maintains the foot and stops it from dropping. Rigid AFOs can help avoid contractures when worn at night. A more energy-efficient gait is offered by AFOs. Another foot brace is the functional foot orthosis (FFO). To reduce stress in the knee, ankle, or foot, an FFO realigns the foot's joints and bones. Individuals could need one FFO or two FFOs [14].

In India, foot braces are commonly used for talipes equinovarus, commonly known as clubfoot. It is a congenital abnormality in which the afflicted foot looks internally rotated at the ankle, the foot points down and inward, and the soles of the feet face each other. Earlier, patients who were treated with surgeries directly, like a posterior release, plantar fasciotomy, or extensive combined release, met with complications. Non-surgical treatments, which include stretching and casting via the Ponseti method, have better outcomes [15]. Once realigned, the foot must be maintained in the same shape via clubfoot braces. Since this treatment demands utmost adherence to the braces, it has become a disadvantage in terms of adaptation to the needs of a growing child. The design and reforms made by engineers, designers, and people in medicine have greatly benefited these patients. New designs ensure better adherence by allowing a range of freedom in the child’s movement with added comfort. The notable braces are the Kessler braces, Horton click braces, Dobb’s dynamic clubfoot braces, the brace made under the supervision of Dr. Posenti, and the Mitchell brace [16].

Researchers at Christian Medical College, Vellore, India, and the Indian Institute of Science, Bangalore, jointly designed a brace called 'Padma Pada' with multiple sensors placed in the soles of the shoes. These sensors assess the wear time every hour for three months and act as a compliance monitoring device. Its revolutionary design assures child adherence by granting them multiple degrees of freedom and enhanced lower-limb mobility [17].

Crutches

A crutch is a long stick with a crosspiece at the top that a person with an injury or handicap uses as a support under the armpit or after a non-weight-bearing surgical procedure. They are vital in the short-term and long-term management of orthopedic and neurologic injuries. This aid is designed to expand an individual’s base of support. There are various walking patterns that individuals using crutches can employ. They include the two-point, three-point, four-point, and swing-to-gait [18]. There are three main types of crutches: axillary crutches, forearm crutches, and platform crutches [19,20].

Axillary crutches are utilized by placing the tube beneath the axillary cavity and grasping the handle below the tube in a parallel manner. The crutches should be positioned approximately 5 cm below the axilla, and the elbow should be flexed at approximately 15 degrees [21]. Forearm crutches are commonly employed for prolonged injuries and medical conditions. While they demand greater strength than underarm crutches, they offer better control over your mobility. They are often easier to use on uneven terrain and when navigating staircases [22].

The design of a crutch has undergone significant improvement since the 1900s. Conventional crutches are heavy, bulky, and made from wood. They are hard to use in confined places, can cause slips, and are associated with an increased risk of radial nerve or crutch palsy and carpal tunnel syndrome. The predominant issue linked to the utilization of axillary crutches is the occurrence of axillobrachial arterial complications resulting from pressure exerted by the axillary bar [23]. In some cases, it may cause recurrent upper-limb ischemia. Hence, new and improved designs are made from aluminum, titanium, and carbon fiber, which are lighter. There are ongoing improvements, with new patents emerging consistently since the advent of crutches. Features such as contoured arm pads, improved tips, and better ergonomics are now standard across the range. Companies such as Ergobaum (Ergoactive, Miami, FL, USA) and M+D (Mobility+Designed, Kansas City, MO, USA) provide crutches with an enhanced range of motion with rubber tips for shock absorption with each step. The reduced weight of crutches helps reduce secondary injuries associated with underarm crutches and increases weight-bearing capacity [24].

Prosthetics

Prosthetics is the branch of medicine where replacement body parts are created and fitted onto an individual's face or body to temporarily change their appearance. A prosthesis is a device meant to replace a missing part of the body, typically a limb, or to improve the function of a part of the body [25]. Prostheses for mobility can be broadly divided into two main groups. Upper-limb prostheses include interscapulothoracic prostheses, transhumeral prostheses, transradial prostheses, and partial wrist prostheses [26]. Lower limb prostheses encompass hemipelvectomy prostheses, hip disarticulation prostheses, above-knee prostheses after transfemoral or above-knee amputation, below-knee prostheses in cases of transtibial or below-knee amputation, and Symes prostheses for Symes amputation or ankle disarticulation [27,28].

Designing and creating a working prosthesis is a huge task for inventors and engineers, as they are intricate designs modified according to the needs of individuals. Until recently, individuals with disabilities in India were dependent solely on costly imported prostheses. But recently, researchers at the Indian Institute of Technology (IIT)-Madras, Chennai, TN, launched India’s first polycentric prosthetic knee, named Kadam. It offers advantages over conventional hinge joints as it has multiple rotational axes with increased control over the prosthesis when walking with over 160 degrees of knee flexion. It is five to six times cheaper than its imported rivals [29]. One Indian non-governmental organization (NGO) named Inali Foundation is delivering cheap upper limb prostheses to people who have lost them in an accident or were born without them. Prashant Gade, the brains behind the idea, has been using the latest 3D printing technique and advanced technologies to create a prosthesis that receives signals from the nerves from the amputation site and converts them into electrical signals for the electrical prosthesis to work at will. The Inali Foundation has made prosthetics related to the upper limb, such as elbow joints, wrist joints, and partial hand prostheses [30]. India's first prosthetics and orthotics company, P&O International Pvt. Ltd., based in New Delhi, launched India’s first myoelectric hand, with thumb rotation controlled by the electrical characteristics of muscles. To acquire the appropriate shape and fit of the limb, the creators employ 3D printing technology, which has made it possible to create exact-fitting prosthetics within a few hours of getting the measurement, as opposed to the three days it would take by hand [31].

The socket is the prosthetic’s most critical component. It is handcrafted and skill-dependent, whereas the knee and foot are prefabricated and standardized. Thus, the prosthesis will not provide a complete result if the socket is incorrect. So, a new process called osseointegration has been devised, a difficult technique even for seasoned orthopaedicians. Osseointegration is a radical treatment in which a skeletal link with prostheses is accomplished by the placement of a titanium implant in the amputee’s bone. This establishes an interface that links directly to a prosthetic limb, adding a new dimension to the amputation restoration process. It is expensive but gives promising results by deleting socket issues, thus leaving no scope for skin breakdowns, discomfort, or friction bleeding. The prosthesis can be removed and worn as soon as possible. Direct bone contact provides a better sense of the earth underneath, allowing the amputee to control the limb more intuitively, almost naturally [32].

The way forward with government and NGO initiatives for the disabled

There are many government organizations and NGOs in India that provide free or subsidized musculoskeletal equipment to people with disabilities. These organizations aim to increase mobility and improve the quality of life. The Department of Empowerment of Persons with Disabilities (Divyangjan) in India has established institutes and colleges to provide quality assistance to the disabled, especially concerning locomotion. Table 1 lists Indian institutes, programs, and organizations and their role in the community for the disabled.

**Table 1:** Government institutes, schemes, and other organizations in India that provide rehabilitation solutions for individuals with disabilities

Government institutes	Objectives
National Institute for Empowerment of Persons With Intellectual Disabilities (NIEPID) [33]	Provides quality services for the intellectually disabled, and has various rehabilitation programs along with specially structured training courses for rehabilitation ranging from certificate to postgraduation levels.
Pandit Deendayal Upadhaya National Institute for Physically Disabled Persons (PDUNIPPD) [34]	Serves people with physical disabilities of all ages, aims to alleviate the pain of people with disabilities, and ensures equal opportunities to participate in the community.
Swami Vivekanand National Institute of Rehabilitation Training and Research (SVNIRTAR) [35]	Provides comprehensive medical services to rehabilitate people with disabilities, as well as vocational and postgraduate courses for individuals from various professional backgrounds such as doctors and engineers to specialize in the field of rehabilitation for the physically disabled.
The National Institute for the Empowerment of Persons With Multiple Disabilities (NIEPMD) (Divyangjan) [36]	Serves as a nationwide resource center to empower people with two or more disabilities. Promotes and conducts research to develop models and strategies to meet the needs of people with multiple disabilities.
The National Institute of Locomotor Disabilities (NILD) [37]	Aims to develop human resources to provide aid to people with orthopedic disabilities, and assistance in the fields of reconstructive surgery with supporting tools and equipment. Also serves as the supreme center of information and documentation in this field.
Government schemes and other organizations	Objectives
Artificial Limbs Manufacturing Corporation of India (ALIMCO) [38]	Manufactures and supplies assistive devices, including prosthetics, to people with disabilities. Frequently partners with government initiatives to supply these devices to the needy at discounted rates.
Bhagwan Mahaveer Viklang Sahayata Samiti (BMVSS) [39]	Known as Jaipur Foot, BMVSS provides prosthetics, stirrups, and other aids and devices to people with disabilities. The goal is to increase mobility and autonomy for people who cannot afford time-consuming, complicated, and costly healthcare.
National Handicapped Finance and Development Corporation (NHFDC) [40]	Finances and implements various programs for economic development to rehabilitate persons with disabilities, and supports the purchase of equipment and support tools.
Rashtriya Vayoshri Yojana (RVY) [41]	A scheme that provides assisted-living tools such as walking canes and crutches among others to senior citizens from the lower economic strata.

State Government Welfare Agencies

Many state governments in India have social welfare departments that run programs to distribute free or subsidized assistive devices. Mobility aids like wheelchairs and crutches are sometimes part of these programs. The Ministry of Social Justice and Empowerment has introduced the Swavlamban health insurance scheme to offer affordable health insurance and coverage to disabled individuals, including broader coverage for various assistive or functional devices [42].

Non-Governmental Organizations

Several NGOs across India are working to provide mobility aids and devices to people with disabilities. It is common for these organizations to collaborate with government programs or operate as autonomous entities. Some notable NGOs are Narayan Seva Sansthan, the Inali Foundation, Mukti India, and the International Society for Human Welfare and Rehabilitation (ISHWAR) [43-46].

Exploring challenges in assistive mobility solutions

While Indian companies have made progress in the field of mobility aids and technology, it is essential to recognize the current limitations that hinder the growth and innovation of this sector. The limitations are mainly related to factors such as outdated equipment, insufficient resources, and limited funding, which pose significant challenges to the development and accessibility of advanced solutions for people with reduced mobility. Outdated infrastructure hinders the development of innovative mobility solutions, limiting the options available to people with diverse mobility needs. Shortages of essential resources, including skilled labor, qualified experts, and dedicated research centers, further exacerbate limitations in this sector. Innovation often requires significant financial investment, making it difficult for Indian companies to conduct in-depth research and prototype development [47].

High Price and Limited Availability

Although technological advances in mobility aids have the potential to have an impact, many face challenges. Significant drawbacks mainly stem from high prices and limited availability. Many advanced devices are often priced beyond the affordability of a significant portion of the population. The economic barriers created by these high costs create division, depriving people with limited mobility of the opportunity to benefit. In India, the coverage provided by health or disability insurance plans for mobility aids is often inadequate. Limited financial support from insurance policies adds to the challenges faced by individuals seeking access to essential equipment. The lack of significant financial support from government initiatives hinders efforts to make these devices more affordable and accessible to more people [48].

Technology Support in India

Many regions in India lack full-service centers equipped with qualified technicians to handle routine repairs and maintenance. This limited service infrastructure makes it difficult for users to access quick and effective support when problems arise with their supportive devices. Users have difficulty sourcing genuine parts for their mobility aids, a situation made more complicated by the prevalence of counterfeit or substandard parts, leading to slowdowns. Delay in the repair process further compromises the reliability of repair and maintenance efforts [48].

Closing the Accessibility Gap

While technological advances are remarkable, it is important to recognize the limitations that stem from user experience and accessibility challenges that are significant for people who have limited mobility and may not be familiar with technology. Complicated controls and setup processes can be frustrating, preventing users from taking full advantage of available support features [49]. Therefore, simplifying the user interface becomes imperative to ensure inclusivity and ease of use for all individuals.

Learning Curve

Inadequate or incomplete training programs can deprive individuals of the skills needed to fully utilize the capabilities of assistive devices. The lack of comprehensive, user-friendly training materials aggravates the learning curve, preventing users from exploiting the full potential of their technology. This slow adaptation can lead to frustration and detachment, affecting overall effectiveness in facilitating independent living and mobility [49].

Discussion

The objective of our study is to discuss the various modalities currently available and in use for the rehabilitation of physically disabled people who find movement difficult. This study delves into the diverse modalities utilized to rehabilitate individuals facing physical disabilities that impede movement. The multifaceted nature of physical disabilities necessitates a comprehensive approach toward rehabilitation, encompassing various therapeutic interventions and assistive technologies. Table 2 summarizes the different types of mobility aids explained above.

**Table 2:** The current mobility aids on the market and their benefits owing to recent developments and innovations

Mobility aid	Benefits
Smart wheelchairs	Offers enhanced maneuverability, obstacle detection, and communication features, empowering users with greater independence and control.
Canes with sensory aid	Equipped with sensors and sonar technology, canes with sensory aids assist individuals with visual impairments by detecting obstacles and enhancing navigational awareness.
Walkers and rollators	Provide improved mobility and comfort compared to traditional walkers, offering adjustable height, outdoor functionality, and optional seating.
Foot braces	Supports various foot conditions and aids in gait correction, emphasizing comfort, mobility, and adherence to treatment protocols.
Crutches	Utilizes lightweight materials and ergonomic features to enhance user comfort, stability, and ease of use, minimizing the risk of secondary injuries and improving mobility.
Prosthetics	Offers individuals with limb loss improved functionality, comfort, and affordability. Innovative solutions, such as India's Kadam knee and 3D-printed prosthetics, exemplify efforts to enhance accessibility and affordability of prosthetic devices. Osseointegration is a new treatment modality with better outcomes.

In addition to exploring rehabilitation modalities, our study delves into the landscape of government initiatives and NGOs dedicated to supporting individuals with physical disabilities in India. The concerted efforts of both the governmental and non-profit sectors play a pivotal role in promoting inclusion, accessibility, and empowerment within the disabled community. Our review uncovered a spectrum of government institutions and schemes tailored to address the diverse needs of individuals with physical disabilities across India. Furthermore, government-sponsored rehabilitation centers and vocational training programs aim to equip individuals with the skills and resources necessary for independent living and socioeconomic integration. Through grassroots initiatives, NGOs bridge gaps in providing psychosocial support and protect the rights and dignity of individuals with physical disabilities. Moreover, collaborative partnerships between governmental agencies and NGOs amplify the impact of interventions, fostering synergistic approaches toward inclusive development. The proliferation of new mobility aids offers promising opportunities for enhancing the independence and mobility of individuals with disabilities. However, alongside these advancements come legitimate concerns regarding the safety and reliability of the materials and components used in their construction. The quality and integrity of mobility aids are paramount in safeguarding users from potential hazards and mitigating the risk of serious injuries or accidents [50,51].

In light of our findings, policymakers and practitioners must prioritize the needs and rights of individuals with physical disabilities, ensuring that rehabilitation services are accessible, equitable, and patient-centered. By leveraging the strengths of government institutions and NGOs, India can chart a course towards inclusive development, where every individual, irrespective of ability, has the opportunity to thrive and contribute meaningfully to society.

## Conclusions

This review emphasizes integrating diverse rehabilitation modalities to address the complex needs of individuals with physical disabilities. From traditional physiotherapy techniques to cutting-edge technologies, the spectrum of rehabilitation interventions offers tailored solutions to enhance mobility, functionality, and overall quality of life. To summarize, the study reveals that there is a substantial need for ongoing research efforts and significant progress in the fields of kinematics, dynamics, modeling, and control methodologies, which are important to improve the present, outdated mobility aids. The links between poor daily activity performance, depression, anxiety, and poverty underline the need for comprehensive treatment for those with mobility issues. Providing more focus on the field of research for modified and better mobility aids will enrich the lives of people with disabilities. It will enable them to lead a life of dignity and independence, with the opportunity to earn a livelihood without discrimination and enjoy a normal social life. The added benefits of mobility devices in maintaining independence and control produce positive attitudes in their users. The government must also increase its capabilities in making public places and services accessible to disabled people. 
